# Antimicrobial Resistance Surveillance: Data Harmonisation and Data Selection within Secondary Data Use

**DOI:** 10.3390/antibiotics13070656

**Published:** 2024-07-16

**Authors:** Sinja Bleischwitz, Tristan Salomon Winkelmann, Yvonne Pfeifer, Martin Alexander Fischer, Niels Pfennigwerth, Jens André Hammerl, Ulrike Binsker, Jörg B. Hans, Sören Gatermann, Annemarie Käsbohrer, Guido Werner, Lothar Kreienbrock

**Affiliations:** 1 Department of Biometry, Epidemiology and Information Processing, WHO Collaborating Centre for Research and Training for Health in the Human-Animal-Environment Interface, University for Veterinary Medicine, 30559 Hanover, Germany; sinja.bleischwitz@gmail.com (S.B.); tristan.salomon.winkelmann@tiho-hannover.de (T.S.W.); 2 Department of Infectious Diseases, Division of Nosocomial Pathogens and Antimicrobial Resistances, Robert Koch Institute, 38855 Wernigerode, Germany; pfeifery@rki.de (Y.P.); fischerma@rki.de (M.A.F.); wernerg@rki.de (G.W.); 3 National Reference Centre for Multidrug-Resistant Gram-Negative Bacteria, Department Medical Microbiology, Ruhr-University Bochum, 44801 Bochum, Germany; niels.pfennigwerth@rub.de (N.P.); joerg.hans@rub.de (J.B.H.); soeren.gatermann@rub.de (S.G.); 4 Division of Epidemiology, Zoonoses and Antimicrobial Resistance, Department Biological Safety, Federal Institute for Risk Assessment, 12277 Berlin, Germany; jens-andre.hammerl@bfr.bund.de (J.A.H.); ulrike.binsker@bfr.bund.de (U.B.); annemarie.kaesbohrer@bfr.bund.de (A.K.)

**Keywords:** One Health, molecular surveillance, cluster analysis, data collection, *Enterobacteriaceae*, multidrug resistance, carbapenem, colistin, *mcr-1*

## Abstract

Resistance to last-resort antibiotics is a global threat to public health. Therefore, surveillance and monitoring systems for antimicrobial resistance should be established on a national and international scale. For the development of a One Health surveillance system, we collected exemplary data on carbapenem and colistin-resistant bacterial isolates from human, animal, food, and environmental sources. We pooled secondary data from routine screenings, hospital outbreak investigations, and studies on antimicrobial resistance. For a joint One Health evaluation, this study incorporates epidemiological metadata with phenotypic resistance information and molecular data on the isolate level. To harmonise the heterogeneous original information for the intended use, we developed a generic strategy. By defining and categorising variables, followed by plausibility checks, we created a catalogue for prospective data collections and applied it to our dataset, enabling us to perform preliminary descriptive statistical analyses. This study shows the complexity of data management using heterogeneous secondary data pools and gives an insight into the early stages of the development of an AMR surveillance programme using secondary data.

## 1. Introduction

The emerging threat of antimicrobial resistance (AMR) has been declared by WHO [1] as “one of the top ten global public health threats facing humanity”. An infection with multidrug-resistant (MDR) pathogens is harmful and dangerous to the patient affected, and the AMR problem in general is deemed an arm’s race between the development of new antimicrobial agents and the resistance mechanisms. Multiple factors drive the spread of resistances, like wider use of antibiotics in general [2,3], false or inappropriate prescription of antibiotics [4], and the (over-)use of antibiotics in animals and humans [5]. 

Therefore, the establishment of a sustainable monitoring and surveillance system (MOSS) to follow the development of AMR is crucial to understanding the drivers of AMR spread and consequences on AMR and to developing new strategies for a sustainable AMR reduction. A lot of investigations attempt AMR MOSS on a national [6,7] or international [8,9,10] level. These systems summarise a lot of efforts in harmonising phenotypic results from the laboratory perspective as well as from the population perspective to have a representative insight into the recent AMR situation (in a country/region). These systems, however, often lack meta information, which was confirmed by a recent study on salmonella genomes, which found that minimum metadata (country, year, and source) is frequently lacking [11]. 

The phenotypic resistance pattern, which is used in other studies [12,13], provides only limited information on the relationship between resistant bacteria, which justifies the use of methods for genotyping like whole genome sequencing (WGS), which provides data as complementary information. For example, chromosomal- and plasmid-located resistance genes can yield improved predictions of transmission pathways and probabilities. On the other hand, other systems (such as specific research studies) may have complex data structures thar incorporate microbiological and WGS information in detail but are often based on limited and biassed data collection, which impedes cross-sectoral evaluation.

MOSS systems, in general, systematically collect and analyse data of public health relevance. While monitoring systems focus on the documented observation of defined measures only, surveillance systems aim towards the prevention or intervention of a public health issue [14]. However, the use of secondary data in a MOSS may result in different or even missing meta information, which hinders the straightforward interpretation of data on the target population and consequently limits the interpretation for AMR transmission between groups or even biases the results. MOSS often includes data from various sources, which needs to be appropriate according to the system’s objectives. The amount and quality of meta data will therefore strongly influence the processing of the information and the outcome of the data analysis. This especially refers to the interpretability of the data [15]. While an AMR MOSS system does not work without high-quality resistance data, it can also be assumed not to be put in an epidemiological or public health context without the high-quality meta data associated with it.

Our study aims to develop an AMR MOSS that incorporates monitoring information from different sectors of AMR, which are the human-, animal-, and food-perspectives of AMR, and sector-specific backgrounds, considering its relations and interactions towards a One Health evaluation to feed into recommendations for public health. To achieve our surveillance system, it therefore consists of the meta data, the phenotypic resistance data, and the molecular genetic resistance information, which should be open to a One Health perspective.

To pilot a One Health approach, this study focuses on carbapenem-resistant *Enterobacteriaceae* (CRE) as an important example for both public (human) and animal health. These carbapenemases are frequently plasmid-encoded and can be transferred between bacterial species, and infections with CRE are associated with high mortality [13]. Treatment options for CRE and carbapenemase-producing *Enterobacteriaceae* (CPE) infections are limited, and polymyxins are often the only alternative [16]. A cross-resistance between CRE and added resistance against polymyxins is therefore especially threatening to the patient and to public health in general. In contrast to carbapenems, colistin is widely administered in livestock (e.g., in Germany), resulting in the dissemination of colistin-resistant bacteria in the animals and the food chain and transmission to humans [16]. Therefore, carbapenems and polymyxins are on the WHO’s list of critically important antibiotics. This study uses data from the German research project “GÜCCI”, a genome-based surveillance project for transmissible colistin and carbapenem resistance in Gram-negative bacteria. We aim to develop strategies to join clinical and epidemiological information and define the requirements for data analysis.

The following paper elucidates the process of data processing for the development of an AMR MOSS. In a step-by-step approach, we show how we define and categorise different variables within the One Health setting. The harmonised data is finally analysed to extract a first glimpse of the One Health situation for transferable colistin and carbapenem resistance in *Enterobacteriaceae* in Germany.

## 2. Results

### 2.1. General Data Collection

Data collected for this study originated from different backgrounds: human samples from hospital care, laboratory samples from pre-existing research studies, animal samples from routine screenings, and food samples from routine monitoring studies.

The data was assessed and harmonised as previously described. As shown in Table 1 and Table 2, we assessed the data individually based on the data provider (RKI, BfR, and RUB) and the data source (bacterial isolates from humans, animals, and food), respectively. Heterogeneity between the data providers becomes apparent. All isolates had been subjected to phenotypic AMR tests (Table 1), but tested antimicrobials and/or corresponding breakpoints and subsequent classifications varied between data providers and data sources, which are shown in Table 2. All results of the antimicrobial resistance testing are shown in the Supplementary Materials (Table S3).

Data selection between providers differed due to the purpose of the primary investigation at each institution. Human patients were sampled due to AMR suspicion, and bacterial isolates were sent to our project partners’ laboratory only if they displayed characteristics of special interests. Animal and food samples are subjected to regular monitoring and further analysed by the respective partner laboratories. All of the data included here was preselected for colistin or carbapenem resistance.

Table 1 shows an overview of the variables that remained for data analysis after harmonisation. If unavailable data exceeded a certain percentage or was incomparable to variables from other datasets, the entire variable was not used for further data analysis.

### 2.2. One Health Use Case “Colistin-Resistant E. coli Isolates”

For a comparative One Health data analysis with data from all sources, variables existing in all respective sources should be used to ensure comparability. For example, the phenotypic resistance profiles of isolates can only be compared if all isolates have been tested for each antimicrobial in question. Thus, a thorough data assessment, as shown above, was necessary before further data analysis. Table 2 shows the number of isolates tested for each antibiotic; the number of resistant isolates is documented in the Supplementary Materials (Table S3).

**Figure 1:**
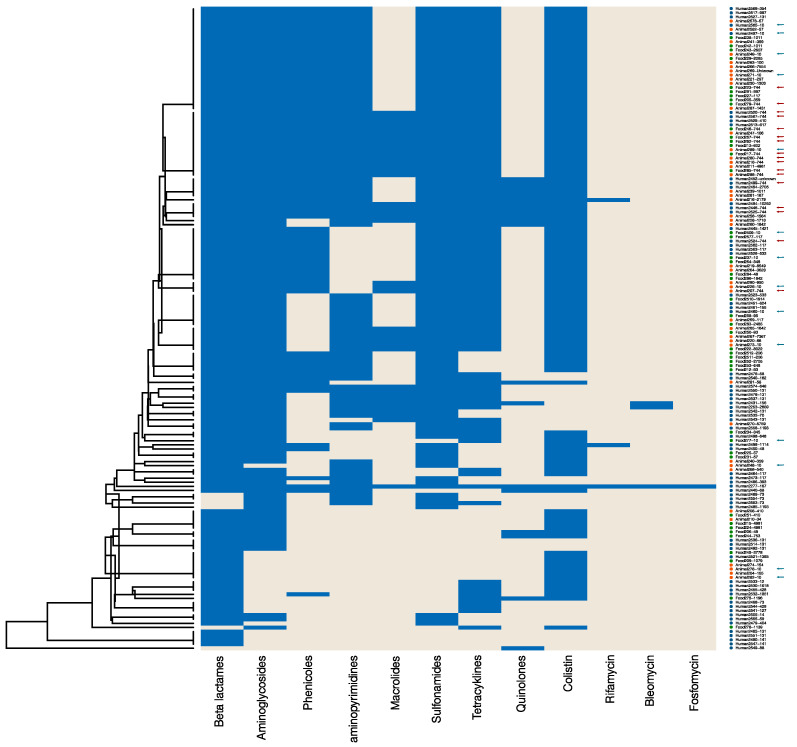
Heatmap and cluster analysis of genetic resistance of 155 *E. coli* isolates with phenotypic colistin resistance. The heatmap (positive and negative findings per antimicrobial class) and a dendrogram were calculated using the Jaccard coefficient and centroid method, ranking the dendrogram values between 0 and 1. Resistance determinants were categorised in groups according to the antimicrobial class to which they mediate resistance (blue). *E. coli* isolates from human patients are highlighted with a blue dot, animal isolates in orange, and food isolates in green. Isolates of the most frequent sequence types ST744 and ST10 in this collection were highlighted with a red arrow and with a blue arrow, respectively.

In the following analyses, we selected *E. coli* isolates displaying phenotypic resistance to colistin exemplarily. Resistance to ertapenem and imipenem was detected in a very low number among isolates from animals and food (Table 2); thus, we chose colistin resistance as the determining factor for this exemplary case study. We examined the genomic data of these isolates as an analytic case study. Isolates with neither PCR nor WGS results were not included. In the end, 155 colistin-resistant *E. coli* isolates (67 human, 44 animal, and 44 food) remained for analysis. A total of 187 resistance determinants were documented, of which some encode for resistance to the same antibiotic class. Therefore, we categorised the information into groups based on the antibiotic class to which the gene mediates resistance using RefGene [17]. Included in the antibiotic classes and single substances were beta-lactams, aminoglycosides, phenicols, diaminopyrimidins, macrolides, lincosamides, sulfonamides, tetracyclines, quinolones, colistin, rifamycin, bleomycin, streptothricin, and fosfomycin, respectively. Genetic resistance analysis was performed by cluster analysis to get a first insight into putative relatedness between isolates. A heatmap (positive and negative findings per antimicrobial class) and a dendrogram were calculated using the Jaccard coefficient and centroid method, ranking the dendrogram values between 0 and 1. We included the multi-locus sequence type (MLST) beside the isolate ID (Figure 1).

Although all 155 isolates were resistant to colistin, including 99 isolates with the resistance gene *mcr-1*, 14 isolates with *mcr-1-like,* and 9 isolates with *mcr-1.2*, interestingly, 32 human isolates and 1 animal isolate with colistin resistance did not display any documented *mcr-like* gene. Conspicuously, 10 of these 32 human isolates belonged to sequence type (ST) 131; this clonal lineage did not appear in *mcr*-positive isolates. Furthermore, some genotypic resistances often appear in combination, such as resistance to beta-lactams and aminoglycosides and resistance to sulfonamides and tetracyclines, throughout all isolate sources. STs within the collective were diverse, with most appearing individually or in very low numbers. However, *E. coli*-ST744 (n = 17) and *E. coli*-ST10 (n = 14) were observed in all three isolate sources.

To demonstrate the variation in resistance profiles, the Jaccard distances to the isolate with the maximum amount of resistance were calculated. With this, distances close to 0 may be interpreted as multidrug-resistant, while distances close to 1 indicate isolates with a high degree of susceptibility. These descriptive measures were calculated by the data source, data supplier, and region in which the isolate was detected (Table 3).

Descriptive statistics of human, animal, and food data show similar results between the sources of the isolates (Table 3). This supports the cluster analysis of human, animal, and food isolates clustering together (Figure 1) and suggests the close genetic relatedness of resistant isolates from different sources and/or the transmission of AMR determinants between sources.

Comparing the distances between data origins within the collective reveals no statistically significant differences between the three data suppliers. However, within the collective under study, RUB isolates appear slightly distinct from RKI and BfR data, which is probably due to the low number of isolates in this collective, which were also mostly pre-selected for carbapenem resistance (n = 2). RKI isolates are from previous research studies, pre-selected for colistin resistance. BfR samples are gathered from routine screenings; however, in our study, they were pre-selected for colistin resistance. The lack of differences visible in the outcome of this calculation suggests that the reason for data collection might have a minor impact only on the genetic profile of the isolates.

Furthermore, we compared the genetic distances of the isolates by region. For this, isolates were categorised by German federal state and subsequently separated by region “North” (i.e., Bremen, Hamburg, Lower Saxony, Mecklenburg-Western Pomerania, and Schleswig-Holstein), “East” (i.e., Berlin, Brandenburg, Saxony, Saxony-Anhalt, and Thuringia), “South” (i.e., Baden-Wuerttemberg and Bavaria), and “West” (i.e., Hesse, North Rhine-Westphalia, Rhineland-Palatinate, and Saarland) (see also Supplementary Table S4). Again, no statistically significant difference was detected between the regions.

Generally, with an average coefficient of variation (CV) of around 28%, we observe a moderate variance of the resistance patterns on the population level, but also a large range of distances between the isolates, suggesting a high genetic diversity in the resistance profiles. In a One Health AMR MOSS such as this, this was to be expected and additionally highlights the importance of epidemiological data analysis in combination with genome data analysis.

## 3. Discussion

This investigation elucidates the process of data harmonisation on the example of a One Health AMR surveillance programme, which is based on the secondary use of data from diverse origins and sources. While there are guidelines for general secondary data use processes, including harmonisation processes for human data in epidemiology [18], these processes are rare in monitoring laboratory data. However, following [18], the use of secondary data always depends on an individual use case and its related purpose. Here we showed in multiple instances how a harmonisation process may take place, with which heterogeneous data may be pooled.

Molecular monitoring systems generally collect data and usually perform data analysis, such as cluster analysis, based on genomic information alone [19,20,21]. Data specifically generated for a MOSS suffices for this type of analysis; however, in our case, secondary data requires additional steps. Secondary data, as opposed to primary data, usually does not include the possibility of gathering all the information from each data source and therefore requires harmonisation.

Harmonisation of secondary data is therefore crucial in a One Health MOSS due to all the restrictions presented here. These restrictions make it difficult to set up an exclusive One Health system, which has also been extensively discussed [22]. Even if working with a catalogue, a predetermined survey, or any other attempt at categorising data from various origins, different interpretations of those catalogues may occur and therefore lead to biases. Working with data without catalogues or surveys specifically made for them (i.e., secondary data) makes this more difficult because harmonisation aids like catalogues are developed after the data is collected.

Catalogues for this project were customarily developed. These catalogues, however, were designed specifically for this study and might need adjustments if additional data is collected in the future. However, a use case based on specific scientific hypotheses to run the MOSS may be drafted. Therefore, to ensure the plausibility of the data, different steps in constructing the One Health MOSS have to be discussed. These are the quality of the information, the use cases defined, and the epidemiological outcome related to the MOSS. In addition, political and socioeconomic influences, such as, for example, data privacy issues, were not included here but finally have to be discussed as well.

### 3.1. Intrinsic and Extrinsic Quality of Information

During plausibility control of the data, it becomes apparent that differences between the data collection methods exist due to the different purposes of our partner institutions. However, even if data are of high quality for their original purpose, for the matter of a One Health system, differences are evident, which is also a multi-faceted issue that exceeds epidemiological research. Therefore, an intrinsic quality and an extrinsic quality (for use in One Health) must be distinguished here.

Reducing extrinsic data quality differences, like standardising resistance evaluation norms, has already been extensively discussed [23,24]. The same is true for data quality in terms of the amount of information. As an example, while PCR data is pre-selected for previously determined genes of interest, WGS data contains all documented resistance determinants. Protocols for laboratory methods differ based on different standards and requirements of human or veterinary medicine, and experimental conditions vary between locations, as well as bioinformatic pipelines, AST plates, etc. This is in line for international systems as well, like the WHO-GLASS MOSS [8], which disregards the AMR interpretation rules as well. These differences in data collection are fine from a clinical perspective. However, from an epidemiological perspective, this often makes data harmonisation a challenge and also leads to a substantial loss of information.

All in all, the processes applied lead to a degree of different representativeness of the resistance situation in the different populations of the associated One Health pillars. This is due to the pre-selection of the isolates in some subsets before colistin resistance testing, although the number of isolates included is substantial (n= 2583 in total). However, the number of human isolates exceeds the number of animal and food isolates greatly, which causes a “One Health imbalance”.

Due to the different reasons for data collection, the isolates were tested for AMR under different circumstances, which influences the outcome of the analysis. Clinical isolates, for example, often display a great variety of AMR because testing for AMR is performed after antibiotic treatment. Another example is the routine AMR testing of food and veterinary samples. Some antimicrobials, such as carbapenems, were not used in veterinary medicine in Germany, which may explain the very low amount of carbapenem resistance in animal and food isolates. Colistin, on the other hand, is widely used in livestock animals, particularly in poultry farms [25,26], but is a last-line antimicrobial in human medicine. However, in human clinics, colistin is tested and considered for treatment (if ever) only when/after carbapenem resistance has been confirmed [27]. This was also the rationale for selecting colistin resistance as the determinant for the analytic case study, to ensure that results were not falsified due to this type of data selection.

### 3.2. Use Cases Defined

The manifold differences within the isolate-collection indicate that the data presented here is neither a representative sample from an overall target population nor a register of all relevant cases, which is a typical pattern in classical MOSS [8,19,28]. Therefore, the meta data included in the MOSS as well as the procedures for constructing the combined data have to be considered to develop use-case-based analyses.

After the harmonisation process and joining the data, the overlap of the remaining information is considerably smaller in our example. The data can, however, be used to draw conclusions and suggestions related to selected parts of the population of isolates. In order to analyse secondary data for passive surveillance, two previous steps are required: data harmonisation and data selection. While data harmonisation was not possible for laboratory analyses in the past, informed data selection in combination with post-hoc meta data requires more effort but allows for a more thorough analysis, which is necessary when working with secondary data pools. Data selection based on a specific research question also allows for more diverse usability.

### 3.3. Antibiotic Resistance Outcome

The use of different laboratory methods by providers and by sources resulted in different AMR information as outcome variables or, more generally, as AMR patterns. This information is complex, like the phenotyping of different components and genotyping of different resistance determinants and virulence factors, which are identified with different technologies and methods. Therefore, the use of multivariate analysis methods like cluster analysis techniques will be influenced as well, especially if the AMR outcome is different. However, cluster analyses can give an insight into the putative genetic relatedness of isolates, as shown in previous studies [20,29], but they also require at least harmonised or plausible comparable AMR outcome data. Our exemplary cluster analysis (see Figure 1) revealed two *E. coli* sequence types, ST10 and ST744, that appear in larger numbers within our selected collective of colistin-resistant *E. coli* isolates from humans, animals, and food. Both lineages belong to pathogenic *E. coli* with increasing importance and have been documented in various global outbreaks [30,31,32,33]. ST131, which was present in isolates without *mcr-like* resistance determinants, is another high-risk clone [34] with the potential to spread colistin resistance independent from plasmids.

Combining specialised sets of genetic and phenotypic resistance information and joining it with meta data and bacteria typing data is therefore the required next step to do a One Health analysis. This shows that evaluating genetic data separately lacks conclusive evidence for a comprehensive interpretation in an epidemiological context due to the heterogeneity of missing values in output variables.

The combination of genetic information with meta data, with consideration of data harmonisation and selection, brings a unique opportunity for One Health assessments. While the overall effort necessary with these types of data analysis is higher, the cost-benefit ratio also shows a significant advantage: results from larger-scale One Health analysis using these methods could potentially be used for public health recommendations. However, the number of isolates that can be evaluated together and presented here is low, so we cannot predict the outcome of large-scale data analysis. Nonetheless, our general approach on how to handle heterogeneous secondary data pools is applicable for other AMR MOSS. The strategy presented here may also be applicable to other research areas evaluating heterogeneous secondary data pools.

## 4. Material and Methods

### 4.1. Data Acquisition

This study utilised secondary data on AMR gathered by the responsible institutions in Germany as a pilot exercise for a secondary data use approach. Data from bacterial isolates was provided in the scope of the German research project “GÜCCI” by the National Reference Centre for Multidrug-resistant Gram-negative Bacteria at Ruhr-University Bochum (RUB), by Robert Koch Institute Germany (RKI), and monitoring data by the German Federal Institute for Risk Assessment (BfR).

The data is pooled from various, heterogeneous origins that have been collected from 2007 to 2020, i.e., before the Corona crisis changed habits and processes, both in sampling as well as in the priorities within laboratory work. By identifying and framing procedures for combining human, animal, food, and environmental data, we wanted to develop an integrated One Health AMR MOSS. It is important to note that the data was not collected specifically for this study (i.e., secondary data), which aims to gain insight on the results of AMR data in retrospect with joint data analysis using harmonisation strategies, plausibility checks, and basic epidemiological analysis, respectively. The heterogeneity of the data is part and implication of the One Health approach we are attempting here.

In detail, the data analysed here has been collected from clinical samples, screening samples, or past AMR research studies. For this, the RUB collected and analysed carbapenem- and colistin-resistant isolates from hospitalised patients throughout Germany. These isolates were screened for the presence of carbapenemase genes and plasmid-mediated colistin resistance genes (*mcr*). The RKI analysed isolates of special interest, i.e., carbapenem-resistant isolates from routine sampling in hospitals and specific research studies that require in-depth laboratory testing. These samples originated from different sources, mainly humans, but rarely from food or the environment. Colistin resistance in human clinical isolates is not routinely determined and assessed in all laboratories. Therefore, selected sentinel laboratories that routinely determine colistin susceptibility in human clinical isolates have sent isolates with colistin resistance for confirmation and further analyses to the RKI since 2016. The BfR provided data on colistin-resistant isolates from routine screenings and in-depth laboratory analyses for AMR of livestock, food, and environmental samples.

In order to be assessable within the means of our study, the data collected from all project partners is subjected to plausibility checks. All corresponding laboratories perform high-standard protocols for sample or isolate analysis, but meta data is often unavailable or sparsely documented and is influenced by the objectives of primary investigation and data collection. We receive the data as a collective dataset from our partners; however, each piece of information about individual isolates requires a thorough examination for usability. Measured values (e.g., MIC) are double-checked, and related variables are compared for plausibility with clinical breakpoints for resistance determination.

In summary, we evaluate clinical data from hospitalised patients, animal data from livestock husbandry, and food-related data from retail samples routinely screened for AMR-carrying bacteria. Isolates were included in this study if they either displayed a phenotypic resistance to meropenem and/or colistin or contained previously published resistance determinants for carbapenems and/or colistin.

### 4.2. Data Structure

The data collection for resistant isolates here is from different providers with different sources (human, animal, and food). Therefore, they use different laboratory methods for resistance determination and collect different meta data linked to the isolates. Isolates from human patients and animals were from various (clinical) materials, such as blood, urine, or stool/faeces/caecal samples. Isolates from food were collected by enrichment- or cultivation-dependent approaches investigating parts of the food matrix. All isolates were identified using standard laboratory methods and then subjected to phenotypic resistance analysis by either broth microdilution or agar disc diffusion, evaluated according to internationally standardised norms (CLSI or EUCAST). Molecular analyses included PCR- or WGS-based screening for different resistance genes, with a focus on plasmid-mediated colistin resistance mediating genes (e.g., *mcr-1*) and carbapenemase-encoding genes (e.g., *bla*_VIM-1_).

For setting up a surveillance system, we categorised the variables of the different origins into four general data pillars, i.e.,

- meta data, which contains epidemiological and clinical information about the original samples
- bacterial typing data, which contains information about the identification and differentiation of bacterial strains
- phenotypic data, which contains information related to phenotypic resistance
- genotypic data, which contains genetic resistance information.

These four pillars will establish the basis for a surveillance system. An overview of all these data items by pillar is shown in the target structure for harmonisation in Table 4.

Some of the transferred variables were identical in each dataset; others were hardly directly comparable (like laboratory methods) or were simply unavailable/unattainable (Table 4, usability comment). The minimum required information for isolates analysed in this study was, aside from the aforementioned AMR: date of sample collection, type of sample source (human, animal, or food), and bacterial species. Aside from identical variables, some information about the isolates was similar between sources, but required additional processes to develop an interface between them. While all isolates were subjected to phenotypic and genotypic resistance analysis, laboratory methods differed. Therefore, the data or its interpretation needed to be harmonised to suit a One Health assessment.

However, other variables remained disjunctive, which goes in line with covering different sectors. Sample matrices, for example, were different between human, animal, and food data. Any food item was categorised according to official German food regulations and followed a strict definition with official catalogues (e.g., type of meat, fresh or frozen). Animal samples were classified by animal species and also by facility type (e.g., breeding facility, fattening facility, slaughterhouse) and isolate matrix (e.g., dust/skin samples, face samples). Both animal and food samples underlie strict rules for definition and categorisation by the German food- and veterinary surveillance and monitoring [35], which do not exist for human clinical samples. Human isolates contained additional information about the age and gender of the patient, whether the patient was hospitalised or not, and isolate material (e.g., blood, urine). All these variables were available in a different context for animal or food samples (sex and age might be included in the definition for the subpopulation sampled, only healthy animals were sampled, and food was derived from healthy animals only). Therefore, this data does not need to be/cannot be harmonised unless separated by data source (human, animal, or food) first.

All isolates were subjected to AST by either broth microdilution (BMD) or agar disc diffusion (ADD). Phenotypic AMR data contained the measured values (zone diameter or minimal inhibitory concentration (MIC)) and interpretation according to the respective breakpoints or epidemiological cut-off values. Both methods used in this study varied regarding the use of automated systems (such as Vitek 2, bioMérieux Inc., Nürtingen, German [36]) or manual BMD procedures. For MOSS development, we considered the measured MIC or zone diameter (ZD) values. The interpretation of the results into “resistant” (R), “intermediate/susceptible increased exposure” (I), and “susceptible” (S) depended on the norms applied. The Clinical & Laboratory Standards Institute (CLSI) established different clinical breakpoint values than the European Committee on Antimicrobial Susceptibility Testing (EUCAST), which are additionally frequently updated and adapted to the current situation. EUCAST also provides epidemiological cut-off values (ECOFFs), which are used for regular monitoring of health animal populations and products thereof. The discrepancy between those two guidelines alone impacts resistance interpretation [37]. These norms are applied depending on whether the data is evaluated according to a clinical/therapeutic inquiry or whether the data is assessed for a public health observation.

### 4.3. Data Quality and Harmonisation Methods

Based on the original data structure shown in Table 1, data harmonisation is crucial for the development of a surveillance programme based on secondary data. Previous studies showed the importance of data harmonisation as well [24]. Here, we elucidated that process in a step-by-step approach. Data quality assessment for this study refers to the completeness, accuracy, plausibility, and precision of documentation.

After we assessed the structure of the raw original data, we defined a target structure for the monitoring system within the concept of the four main pillars described above. The variables observed were classified as

Pillar Meta Data
  - Date of sampling
  - Date of isolation
  - Regional Code
  - City
  - State
  - Data Source
  - Reasons for data collection
  - Sample location origin (with separated catalogues by source)
  - Matrix/Material (with separated catalogues by source)

Pillar Bacterial Typing
  - Identified bacterial species
  - MLST
  - Plasmid replicons (data not shown in the result section)
  - pMLST (data not shown in the result section)
  - Determination method of bacterial species identification

Pillar Phenotypic AMR Data
  - Method
  - Interpretation norm
  - Tested AM
  - Measured value (of MIC/ADD)
  - Interpretation result

Pillar Genomic AMR Data
  - Method
  - Resistance determinants

For this study, data was harmonised manually following the above structure by in-depth consultation with the project partners about their respective data. However, the generic components of this process can be used as a framework for future activities.

Metrically scaled data, here the date of sampling and the patient’s age, can be directly compared between different data sources. For nominal data, usually catalogues are a commonly used tool to classify data into categories. Officially standardised catalogues exist for some variables only. These are, for example, the German federal coding matrices for food monitoring [38], the list of municipal information systems, and the National Center for Biotechnology Information (NCBI) Reference Gene Catalogue [17]. For most of the variables defined above, no formal catalogues exist. Therefore, we generated custom catalogues to categorise variables into comparable groups to guide the harmonisation process (see Supplementary Tables S1 and S2).

Bacterial species were identified on a lab-dependent basis. However, usually a confirmation step is performed in each laboratory [39]. Therefore, we considered whether a defined bacterial species changed after a confirmation run. The bacterial species are documented in Supplementary Table S2.

Within the harmonisation process, several challenges have to be addressed. First, incomplete datasets with missing observations may appear. This cannot be influenced directly within secondary data in hindsight, and therefore, missing data is a reoccurring issue that needs to be addressed and documented to optimise the data usability. Generally, we divided the missing data into the following categories:

- data not documented
- data documented insufficiently or with low quality and
- data documented as wrong or not plausible.

The reasons for missing data are manifold. One reason is that observations of variables may simply not have been acquired. Documentation of meta data from human hospitalised patients, for example, usually follows individual adapted protocols per hospital and therefore is not standardised. Additionally, hospital data underlies the data privacy restrictions, so using personal information directly is limited or even omitted due to limited resources for pseudonymisation or other privacy measures regarding data processing and handling. This is a specific concern for missing meta information, e.g., the subject’s disease indication and other information from the healthcare setting.

First, not-documented data on phenotypic resistance (i.e., “not documented” due to “not tested”) was identified for all data sources used. Test panels for AMR were compared across data sources. For the One Health approach, only antimicrobials tested in every data source remained for combined analyses. Data sources also use different norms to interpret the results, whether an isolate is resistant or not [40,41]. This information could not be harmonised but was kept for further.

Second, data that was acquired but differed in quality was nonetheless included. The amount of information on a variable may vary depending on the data source. For instance, the location of an isolate was reported with different aggregation levels, like a detailed city or a 3-digit postal code only, which stratifies Germany into approximately 100 sub-regions. However, the quality of the regional information differs as well. The location of the origin of an isolate could be interpreted as the location where the sampling took place, the location of the laboratory performing the isolation, or the home or even the birthplace of the patient. The data provided here contained no information in this regard, which will restrict the development of use cases for secondary data.

Third, wrong or not plausible data may occur for various reasons. This especially regards information that has been acquired post-hoc from the raw data. For example, extracting the location origin from only the first three digits of the postal code generally retrieves information on the federal state level. However, in very specific cases, the first three digits of a postal code are shared by two federal states, thus resulting in a possibly false category. If information on the federal state cannot be obtained or concluded from other variables, the category was assigned at random.

Therefore, preliminary and post-hoc data evaluation step-checking for these items is necessary. Especially regarding the use case, i.e., here, the One Health context (view below).

An isolate analysed by WGS was associated with positive or negative findings of resistance genes because a gene not found in the WGS screen is likely not present in the isolate. This means that resistance genes not identified are interpreted as “not apparent”. In addition, based on different bioinformatics pipelines, the denotation of resistance determinants was not standardised beforehand. Therefore, a post-hoc denotation was applied [15].

Similarly, we evaluated PCR data. Resistance determinants discovered by PCR were treated as a positive finding; however, genes not tested by PCR were not compared to WGS information.

### 4.4. Definition of Use Cases for One Health Data Analysis

After individual harmonisation, we combined all the data for the One Health assessment. Therefore, for a One Health analysis, data has to be selected based on a specific research hypothesis, which should be formulated as a so-called use case. However, if data is not feasible for a One Health assessment, it may still be incorporated in analytic case studies.

As an exemplary One Health Use Case here, we evaluated *E. coli* isolates with phenotypic colistin resistance. Within our AMR surveillance system, we focused on discovering putative transmission paths of resistant pathogens, as well as genetic resistance patterns.

### 4.5. Statistics

Statistical evaluations in this study were generally performed with SAS, Version 9.4 TS Level M5 (SAS Institute Inc., Cary, NC, USA). Comparison of data sources and general improvement of completeness and plausibility were done with ordinary data management and data description routines.

Comparing isolates from humans, animals, food, and the environment, as well as AMR patterns, was conducted with classical contingency tables. To generate a multivariate statistical outcome for phenotyping and genotyping, resistance profiles were generated, and we followed the statistical concepts of Anderson et al. and Ruddat et al. [42,43]. In that context, we evaluated resistance determinants based on their respective antimicrobial classes to overcome the discrepancies in data quality. Genetic profiles of isolates were measured by transforming information on resistance determinants into 0–1-vector matrices and then calculated as distance measures using the Jaccard coefficient without prioritising any specific gene. These distances can take on values from 0 (identical distance, i.e., genetic profile) to 1 (no accordance in genetic profile).

With this, we selected one isolate as an “epidemiological reference” based on the genetic profile of resistance determinants and measured distances. This designated isolate contained resistance determinants coding for all documented antibiotic classes. The isolate is a human *E. coli* isolate from a clinical urine sample collected in Bavaria with phenotypic resistance to colistin, meropenem, imipenem, and ertapenem. We used the distance of each isolate to this reference to describe differences in data source and origin, as well as the region of the sample, as parameters. Here, small distances to the reference indicate isolates with multiple resistance genes. In contrast, distances close to one indicate isolates with only a few resistance genes. To check for statistical significance between these groups, the permutation test of Anderson et al. was used [42,43].

In addition, a cluster analysis was conducted hierarchically using the centroid method. Heatmaps and dendrograms were plotted using the R package “ggplot2” (R version 1.4.1717).

## 5. Conclusions

In summary, our study shows that heterogeneous secondary data pools can be used for passive AMR MOSS. However, analysing data requires thorough previous harmonisation and data selection in order to make conclusive interpretations for a One Health assessment. Secondary data pools in published MOSS aim to harmonise data by, e.g., broadening the categories and harmonising based on common information rather than differences. In contrast, our approach is to turn joint data pools into smaller data collectives while considering the differences. Any results presented in this study are preliminary, also regarding the possibility that datasets might be incomplete and additional data might be provided by future collaboration partners.

## Figures and Tables

**Table 1:** Schematic overview of isolates by data origin.

	Provider *		
	RKI	RUB	BfR
Isolates total	353	2147	83
Meta Data			
Data source	353	2147	83
Isolation date	338	2147	83
State	206	2146	0
City	0	2147	68
Regional code	264	1571	0
Matrix	292	2145	83
Sample location origin	212	222	83
Bacterial Typing			
MALDI Bacterial species identification	353	2146	83
WGS Confirmation of bacterial species	127	2146	83
MLST data	282	319	83
Phenotypic Data			
Phenotypic AMR testing	353	2147	83
Genotypic Data			
WGS data	147	222	83
PCR data	353	2147	83

* Providers: National Reference Centre for Multidrug-resistant Gram-negative Bacteria at Ruhr-University Bochum (RUB), Robert Koch Institute Germany (RKI), and the German Federal Institute for Risk Assessment (BfR).

**Table 2:** Frequency distribution of AM-resistance tests, separated by source. Isolates were pre-selected for either colistin or meropenem resistance.

Antimicrobial	Human		Animal		Food	
	n *	%	n *	%	n *	%
Aminoglycosides						
Amikacin	234	9.44	1	1.89	8	15.69
Gentamicin	315	12.71	44	83.02	48	94.12
Kanamycin	270	10.89	11	20.75	22	43.14
Tobramycin	45	1.82	0	0.00	0	0.00
Beta-lactams						
Ampicillin	270	10.89	44	83.02	48	94.12
Aztreonam	45	1.82	0	0.00	0	0.00
Cefepime	45	1.82	12	22.64	15	29.41
Cefotaxime	315	12.71	44	83.02	48	94.12
Cefotaxime/Clavulanic Acid	0	0.00	16	30.19	15	29.41
Cefotiam	68	2.74	44	83.02	44	86.27
Cefoxitin	270	10.89	13	24.53	23	45.10
Ceftazidime	315	12.71	44	83.02	48	94.12
Ceftazidime/Clavulanic Acid	0	0.00	16	30.19	15	29.41
Ertapenem	1889	76.20	12	22.64	15	29.41
Imipenem	2192	88.42	12	22.64	15	29.41
Meropenem	2458	99.15	36	67.92	29	56.86
Mezlocillin	68	2.74	1	1.89	6	11.76
Mezlocillin/Sulbactam	68	2.74	1	1.89	6	11.76
Piperacillin	45	1.82	0	0.00	0	0.00
Piperacillin/Tazobactam	45	1.82	0	0.00	0	0.00
Temocillin	0	0.00	16	30.19	15	29.41
Quinolones						
Ciprofloxacin	315	12.71	44	83.02	48	94.12
Moxifloxacin	45	1.82	0	0.00	0	0.00
Nalidixic acid	270	10.89	44	83.02	48	94.12
Diaminopyrimidins						
Sulfamethoxazole/Trimethoprim	315	12.71	1	1.89	6	11.76
Sulfamethoxazole	0	0.00	43	81.13	44	86.27
Trimethoprim	0	0.00	43	81.13	44	86.27
Macrolides						
Azithromycin	0	0.00	37	69.81	26	50.98
Polymyxins						
Colistin	1570	63.33	53	100.00	49	96.08
Others						
Chloramphenicol	267	10.77	44	83.02	48	94.12
Fosfomycin	45	1.82	6	11.32	14	27.45
Tetracycline	79	3.19	43	81.13	40	78.43
Tigecycline	45	1.82	36	67.92	25	49.02
Oxytetracycline	190	7.66	1	1.89	8	15.69
Streptomycin	191	7.70	11	20.75	22	43.14
Tests total	2479		53		51	

* The total amount of measured resistance (n) varies also due to the different amounts of isolates tested.

**Table 3:** Descriptive measures for Jaccard distances to the “most multi-resistant” isolate” of 154 colistin-resistant *E. coli* isolates by data source, data supplier, and region.

	n	Mean	Med.	Std.	Cv	Min	5%-Perc.	95%-Perc.	Max
data source									
Human	66	0.61	0.54	0.19	30.48	0.23	0.38	0.92	0.92
Animal	44	0.50	0.46	0.15	29.66	0.31	0.31	0.85	0.85
Food	44	0.55	0.54	0.13	24.30	0.38	0.38	0.77	0.85
data supplier									
RKI	71	0.60	0.54	0.18	29.51	0.23	0.38	0.92	0.92
RUB	2	0.42	0.42	0.05	12.86	0.38	0.38	0.46	0.46
BfR	81	0.52	0.46	0.15	28.14	0.31	0.38	0.85	0.85
region									
North	24	0.53	0.46	0.15	28.19	0.31	0.38	0.85	0.85
South	13	0.53	0.46	0.16	29.17	0.38	0.38	0.85	0.85
West	72	0.61	0.54	0.18	29.04	0.31	0.38	0.92	0.92
East	19	0.56	0.54	0.16	29.19	0.31	0.31	0.85	0.85
unknown	26	0.46	0.46	0.10	20.94	0.23	0.31	0.62	0.69
ALL	154	0.56	0.54	0.17	29.76	0.23	0.38	0.85	0.92

Legend: n sample size; mean arithmetic mean; med. median; std. standard deviation; cv coefficient of variation; min minimum; 5%-perc. 5%-percentile; 95%-perc. 95%-percentile; max maximum.

**Table 4:** One Health usability of data.

Variable	RUB	RKI	BfR	Usability Comment
Meta Data				
Date of sampling		✓	✓	Requires harmonisation of documentation
Date of isolation	✓	✓	✓	Requires harmonisation of documentation
Reasons for data collection	clinical isolates with AMR suspicion	Isolates of special interest	Routine screenings/regular monitoring	Evaluable in a One Health context
Regional Code	✓	✓		Requires harmonisation of documentation
City	✓		✓	Requires harmonisation of documentation
Federal state	✓	✓		Evaluable in a One Health context
Source	Human	Human, Food	Animal, Food	Evaluable in a One Health context
Age	✓	✓		For human data only
Gender	✓	✓		For human data only
Sample location origin	✓	✓	✓	differentiated by data source
Matrix	✓	✓	✓	differentiated by data source
Bacterial Typing				
Bacterial species	✓	✓	✓	Requires harmonisation of documentation
Method	✓	✓, with WGS confirmation	✓, with WGS confirmation	differentiated by data provider
MLST	✓	✓	✓	Evaluable in a One Health context
Phenotypic Data				
Method	BMD, Agar disc diffusion,	BMD, autom. AST	BMD, autom. AST	Not evaluable in a One Health context
Evaluation norm	EUCAST	EUCAST	CLSI/EUCAST	Not evaluable in a One Health context
Tested Antibiotics	✓	✓	✓	Requires harmonisation
Interpretation		✓	✓	Requires harmonisation
Genotypic Data				
Method	PCR, WGS	PCR, WGS	PCR, WGS	differentiated by data provider
Resistance Determinants	✓	✓	✓	Requires harmonisation of documentation

Legend: ✓ variable present; RUB National Reference Centre for Multidrug-resistant Gram-negative Bacteria at Ruhr-University Bochum; RKI Robert Koch Institute Germany; BfR German Federal Institute for Risk Assessment; WGS whole genome sequencing; (p)MLST (plasmid) multi-locus sequence typing; BMD broth microdilution; autom AST antibiotic susceptibility testing by automated system VITEK II (Biomérieux, Nuertingen, Germany).

## Data Availability

The data were collected from existing data-bases of the participating laboratories (G.W., A.K. and S.G.) with the understanding that full data would not be transferred to any third party. Therefore, complete data transfer to interested parties is not allowed without an additional formal contract. Data are available to qualified researchers who sign a contract with the project partners. This contract will include guarantees of the obligation to maintain data confidentiality in accordance with the provisions of the European General Data Protection Regulation and its supporting rules in Germany. Currently, there is no data access committee or another body that could be contacted for the data. However, for this purpose, a committee will be formed. This future committee will consist of the authors’ institutions as well as members of the funding institution (the German Minister of Health). Interested cooperative partners who are able to sign a contract as described above may contact Lothar Kreienbrock (lothar.kreienbrock@tiho-hannover.de), Department of Biometry, Epidemiology, and Information Processing, University of Veterinary Medicine Hannover, Bünteweg 2, 30559 Hannover. Data management and analyses were performed using SAS, Version 9.4 TS Level M5 (SAS Institute Inc., Cary, NC, USA), and public available programs using R software. The authors are willing to share non-confidential parts of the programming codes upon reasonable request.

## Supplementary Materials

The following supporting information can be downloaded at: https://www.mdpi.com/article/10.3390/antibiotics13070656/s1, Table S1: Scaling of variables by data source, Table S2: Isolates by sample origin, Table S3: Isolates by genera, Table S4: Number and proportion of phenotypically resistant isolates by antibiotic and antibiotic class, Table S5: Example for data selection within the collective, here 155 *E. coli* isolates displaying a phenotypic resistance to colistin.

## Abbreviations

ADD	Agar disc diffusion
ARS	Antimicrobial Resistance Surveillance
AMR	Antimicrobial resistance
AST	Antimicrobial susceptibility testing
BMD	Broth microdilution
CLSI	Clinical and laboratory standards institute
CRE	Carbapenem-resistant *Enterobacteriaceae*
CPE	Carbapenemase producing *Enterobacteriaceae*
CV	Coefficient of variance
EARS-Net	European Antimicrobial Resistance Surveillance Network
EUCAST	European Committee on Antimicrobial Susceptibility Testing
GLASS	Global Antimicrobial Resistance and Use Surveillance System
MDR	Multidrug-resistant
MIC	Minimum inhibitory concentration
(p)MLST	(plasmid) Multi-locus sequence typing
MOSS	Monitoring or surveillance system
PCR	Polymerase Chain Reaction
STD	Standard deviation
WHO	World Health Organisation
WGS	Whole Genome Sequencing
