# Supplementary material for: Antimicrobial Resistance Surveillance: Data Harmonisation and Data Selection within Secondary Data Use
Source: Antibiotics (Basel). 2024 Jul 16;13(7):656. doi: 10.3390/antibiotics13070656 (PMC11273461; doi:10.3390/antibiotics13070656)
Supplement: Supplementary file 1 [file antibiotics-13-00656-s001.zip › antibiotics-3018012-supplementary.pdf]

antibiotics MDPI

Supplementary Materials

Table S1: Scaling of variables by data source

	Variable	Scaling	Format
**RKI**	Receipt Date	Metric	DD.MM.YYYY<br>YYYY
	Isolation Date	Metric	YYYY<br>Month/YYYY
	State	Nominal	[char]
	Zip Code	Nominal	[num]
	Isolate Matrix	Nominal	[char]
	Species	Nominal	[char]
	WGS Species	Nominal	[char]
	Sex	Nominal	[char]
	Age	Metric	[num]
	Location Origin	Nominal	[char]
	MIC [name]	Ordinal	[num]
	Resistance Determinant	Nominal	[char]
	PCR	Nominal	[char]
	WGS MLST	Nominal	[char]
**BfR**	Isolation Date	Metric	DD.MM.YYYY
	Facility type	Nominal	[char]
	Matrix	Nominal	[char]
	Matrix Catalogue Code	Nominal	[num]
	Species	Nominal	[char]
	MALDI Species	Nominal	[char]
	Producing facilities	Nominal	[char]
	MIC [name]	Ordinal	[num]
	Resistance Determinant	Nominal	[char]
	MLST	Nominal	[char]
	MLST allel type	Nominal	[char]
	PCR	Nominal	[char]
	virulence determinants	Nominal	[char]
	Plasmid Replicons	Nominal	[char]
	pMLST types	Nominal	[char]
**RUB**	Receipt Date	Metric	DD.MM.YYYY
	City	Nominal	[char]
	State	Nominal	[char]
	Zip Code	Nominal	[num]
	Isolate Matrix	Nominal	[char]
	Species	Nominal	[char]
	Age	Metric	[num]
	nosocomial infection	Nominal	[char]
	foreign country anamnesis	Nominal	[char]
	Agar disc diffusion [name]	Ordinal	[num]
	MIC [name]	Ordinal	[num]
	Resistance Determinant	Nominal	[char]
	PCR	Nominal	[char]

Table S2: Isolates by sample origin

Sample Source	Total	Percentage in %
**Animals**		
Pig, Faeces	14	31.82
Poultry, Caecal content	14	31.82
Poultry, Faeces	12	27.27
Calf, Faeces	3	6.82
Poultry, Skin	1	2.27
**All**	**44**	**100.00**
**Food**		
Food from retailer	38	95.00
Not classified by food leg-islation	2	5.00
**All**	**44**	**100.00**
**Humans**		
Rectal	760	30.60
Urine	728	29.31
Wound	258	10.39
Other	223	8.98
Respiratory	206	8.29
Unknown	137	5.52
Blood	79	3.18
Skin	51	2.05
Intraabdominal	38	1.53
Cervix/Vagina	2	0.08
Total	2,482	100.00

Table S3: Isolates by genera

Citrobacter	*amalonaticus*	5	0.23%
	braakii	6	0.27%
	farmeri	2	0.09%
	farmeri/amalonaticus	1	0.05%
	freundii	312	14.09%
	koseri	4	0.18%
	sedlakii	1	0.05%
	spp.	3	0.14%
	werkmanii	1	0.05%
	youngae	1	0.05%
Enterobacter	*asburiae*	9	0.41%
	cloacae	760	34.31%
	hormaechei	2	0.09%
	kobei	3	0.14%
	spp.	2	0.09%
Escherichia	*coli*	684	30.88%
Klebsiella	*aerogenes*	13	0.59%
	oxytoca	268	12.10%
	planticola	2	0.09%
	pneumoniae	251	11.33%
	variicola	18	0.81%
Pseudomonas	*aeruginosa*	143	6.46%
	fulva	1	0.05%
	putida	2	0.09%
Raoultella	*ornithinolytica*	8	0.36%
	ornithinolytica/planticola	1	0.05%
	planticola	1	0.05%
	spp.	1	0.05%
Others			
Alcaligenes	*faecalis*	1	0.05%
Hafnia	*alvei*	5	0.23%
Leclercia	*adecarboxylata*	1	0.05%
Morganella	*morganii*	7	0.32%

Proteus	*mirabilis*	7	0.32%
Providencia	*stuartii*	7	0.32%
Serratia	*marcescens*	50	2.26%

Table S4: Number and proportion of phenotypically resistant isolates by antibiotic and antibiotic class

Antimicrobial Resistance	Human		Animal		Food	
	n	% of tested isolates	n	% of tested isolates	n	% of tested isolates
**Aminoglycosides**						
Amikacin	15	6.41	0	0.00	0	0.00
Gentamicin	58	18.41	11	25.00	4	8.33
Kanamycin	12	4.44	2	18.18	2	9.09
Tobramycin	6	13.33	0	n.t.	0	n.t.
**Beta-lactams**						
Ampicillin	236	87.41	41	93.18	44	91.67
Aztreonam	5	11.11	0	n.t.	0	n.t.
Cefepime	2	4.44	15	100.00	15	100.00
Cefotaxime	162	51.43	22	50.00	28	58.33
Cefotaxime/Clavulanic Acid	0	n.t.	0	0.00	0	0.00
Cefoxitin	172	63.70	0	0.00	5	21.74
Ceftazidime	156	49.52	0	0.00	3	6.25
Ceftazidime/Clavulanic Acid	0	n.t.	0	0.00	0	0.00
Ertapenem	1842	97.05	1	8.33	0	0.00
Imipenem	1869	85.26	0	0.00	0	0.00
Meropenem	1420	57.72	0	0.00	0	0.00
Mezlocillin	49	72.06	1	100.00	4	66.67
Mezlocillin/Sulbactam	0	0.00	0	0.00	0	0.00
Piperacillin	31	68.89	0	n.t.	0	n.t.
Piperacillin/Tazobactam	4	8.89	0	n.t.	0	n.t.
Temocillin	0	n.t.	0	0.00	0	0.00
**Quinolones**						
Ciprofloxacin	134	42.54	27	61.36	34	70.83
Moxifloxacin	0	0.00	0	n.t.	0	n.t.
Nalidixic acid	201	74.44	0	0.00	4	8.33
**Diaminopyrimidins**						
Sulfamethoxazole/Trimethoprim	208	66.03	0	0.00	0	0.00
Sulfamethoxazole	0	n.t.	35	81.40	29	65.91
Trimethoprim	0	n.t.	30	69.77	22	50.00
**Macrolides**						
Azithromycin	0	n.t.	0	0.00	0	0.00
**Polymyxins**						
Colistin	165	10.51	44	83.02	42	85.71
**Others**						
Cefotiam	14	20.59	1	2.27	5	11.36
Chloramphenicol	143	53.56	21	47.73	24	50.00
Fosfomycin	1	2.22	0	0.00	3	21.43
Tetracycline	0	0.00	33	76.74	27	67.50
Tigecycline	1	2.22	0	0.00	0	0.00
Oxytetracycline	33	17.37	1	100.00	2	25.00
Streptomycin	35	18.32	1	9.09	5	22.73

*n.t.: not tested

Table S5: Example for data selection within the collective, here 155 *E. coli* isolates displaying a phenotypic resistance to colistin.

	**Human**	**Animal**	**Food**
**Total Isolates**	67	44	44
**Reason for data collection**			
AMR suspicion case	3		
Special interest	64	44	44
**Meta-Data: Location by Federal State**			
Bavaria	1	1	2
Baden-Wuerttemberg	1	0	9
Berlin	3	0	4
Hesse	5	1	2
Lower Saxony	0	16	3
North Rhine- Westphalia	46	6	10
Mecklenburg Western Pomerania	0	4	4
Saarland	0	0	2
Saxony	1	3	2
Saxony-Anhalt	0	1	4
Thuringia	0	2	0
Unknown	10	0	1
